# Genome-wide survey of parent-of-origin effects on DNA methylation identifies candidate imprinted loci in humans

**DOI:** 10.1093/hmg/ddy206

**Published:** 2018-06-01

**Authors:** Gabriel Cuellar Partida, Charles Laurin, Susan M Ring, Tom R Gaunt, Allan F McRae, Peter M Visscher, Grant W Montgomery, Nicholas G Martin, Gibran Hemani, Matthew Suderman, Caroline L Relton, George Davey Smith, David M Evans

**Affiliations:** 1 University of Queensland Diamantina Institute, Translational Research Institute, Brisbane, QLD, Australia; 2 Medical Research Council (MRC) Integrative Epidemiology Unit, Population Health Sciences, Bristol Medical School, University of Bristol, Bristol, UK; 3 The Institute for Molecular Bioscience, University of Queensland, Brisbane, QLD, Australia; 4 Queensland Brain Institute, University of Queensland, Brisbane, QLD, Australia; 5 QIMR Berghofer Medical Research Institute, Brisbane, QLD, Australia

## Abstract

Genomic imprinting is an epigenetic mechanism leading to parent-of-origin silencing of alleles. So far, the precise number of imprinted regions in humans is uncertain. In this study, we leveraged genome-wide DNA methylation in whole blood measured longitudinally at three time points (birth, childhood and adolescence) and genome-wide association studies (GWAS) data in 740 mother–child duos from the Avon Longitudinal Study of parents and children to identify candidate imprinted loci. We reasoned that *cis*-meQTLs at genomic regions that were imprinted would show strong evidence of parent-of-origin associations with DNA methylation, enabling the detection of imprinted regions. Using this approach, we identified genome-wide significant *cis*-meQTLs that exhibited parent-of-origin effects (POEs) at 82 loci, 34 novel and 48 regions previously implicated in imprinting (3.7^−10^<*P* < 10^−300^). Using an independent dataset from the Brisbane Systems Genetic Study, we replicated 76 out of the 82 identified loci. POEs were remarkably consistent across time points and were so strong at some loci that methylation levels enabled good discrimination of parental transmissions at these and surrounding genomic regions. The implication is that parental allelic transmissions could be modelled at many imprinted (and linked) loci in GWAS of unrelated individuals given a combination of genetic and methylation data. Novel regions showing parent of origin effects on methylation will require replication using a different technology and further functional experiments to confirm that such effects arise through a genomic imprinting mechanism.

## Background

Genomic imprinting is an epigenetic mechanism in which genes are silenced in a parent-of-origin specific manner. The first experimental evidence for genomic imprinting was provided by investigations during the 1980s when researchers failed to produce viable mouse embryos using only the paternal or maternal genome (1). The precise evolutionary mechanisms that give rise to genomic imprinting are unknown. One hypothesis postulates that imprinting provides a mechanism through which maternal and paternal genomes exert counteracting growth effects during development with paternal genes encouraging growth and solicitation of maternal care, even at the expense of the mother’s health, while maternal alleles are oriented toward success of all offspring, who do not necessarily share the same father (2). There is some empirical evidence to support this hypothesis. For example, in contrast to expression of the paternally derived insulin-like growth factor 2 (*IGF2*) gene that promotes cell proliferation, expression of the maternally derived *CDKN1C* and *PHLDA2* genes act as negative regulators of this process (3). 

It is widely accepted that imprinted genes are regulated by *cis*-acting regulatory elements, called imprinting control elements, which carry parental-specific epigenetic modifications such as DNA methylation (4). DNA methylation mainly occurs at the C5 position of CpG dinucleotides and is known to influence transcription (4). Promoter regions of imprinted genes are usually rich in CpG sites and within differentially methylated regions (DMRs) where the repressed allele is methylated and the active allele is unmethylated. Although typical imprinting of a region results in monoallelic expression of the paternal or maternal allele, studies have shown that loci can deviate from this canonical pattern and show differential expression in a parent-of-origin-dependent manner (5,6). 

Multiple studies have shown that imprinted genes affect prenatal growth control, normal brain development and postnatal metabolism (7–10). The monoallelic expression of imprinted loci produces genetic vulnerabilities that can lead to monogenic syndromes. In humans, abnormal imprinting patterns at specific loci can result in genetic disorders such as Beckwith–Wiedemann and Silver–Russell syndromes that primarily affect growth, and Angelman and Prader Willi syndromes which have marked effects on growth and behaviour (11). Evidence is also growing that imprinted genes may play a significant role in complex human traits. Early linkage studies found evidence that genomic imprinting was important in the genetic aetiology of mental disorders such as Alzheimer’s and schizophrenia as well as Type 2 diabetes (T2D) and body mass index (12–14). More recently, large-scale genome-wide association studies (GWAS) have found SNPs within imprinted genes that exhibit parent of origin effects and are associated with traits including age at menarche, breast cancer, basal cell carcinoma or T2D (15–18). 

Given that genomic imprinting appears to play a role in the genetic aetiology of multiple complex phenotypes, identifying novel imprinted genes is of considerable interest. However, the extent to which genes exhibit imprinted expression throughout the human genome is unknown. The number of validated imprinted genes in humans lies somewhere between 40 and 100 according to reviews (19–21), while some databases such as geneimprint (http://www.geneimprint.com/; date last accessed January 10, 2018) and the Otago imprinting database (22) list many more that have yet to be validated. Several methods have been used to identify imprinted loci, including analysis of differential expression between parthenogenotes and androgenotes in mice (23), bioinformatic approaches that look for novel imprinted loci based on genomic features found in known imprinted regions (24), and creating gene knockouts of paternal/maternal alleles in mice (25). More recently, whole genome scans of imprinted regions have been performed using next-generation sequencing technologies to measure differential gene expression between maternally and paternally derived genes using RNA-seq (26–28) or to measure differential methylation with MethylC-Seq (29). Although some of these more recent approaches have been applied to human genomes, the number of studies has been limited and constrained to small sample sizes (27,30,31), thus limiting the ability to reliably detect imprinted genes. 

Imprinted regions in the human genome can also be detected using statistical approaches that model parent-of-origin effects (POEs) of genetic variants on DNA methylation and gene expression. In the presence of imprinting, SNPs affecting DNA methylation (mQTLs) or gene expression (eQTLs) have a different effect depending on their parental origin. In this work, we leverage genome-wide DNA methylation and genotypic data of up to 740 mother–child duos from the Avon Longitudinal Study of Parents and Children (ALSPAC) to identify candidate imprinted loci.

## Results

### Identification of methylation POEs and candidate imprinted DMRs

**Figure 1.**
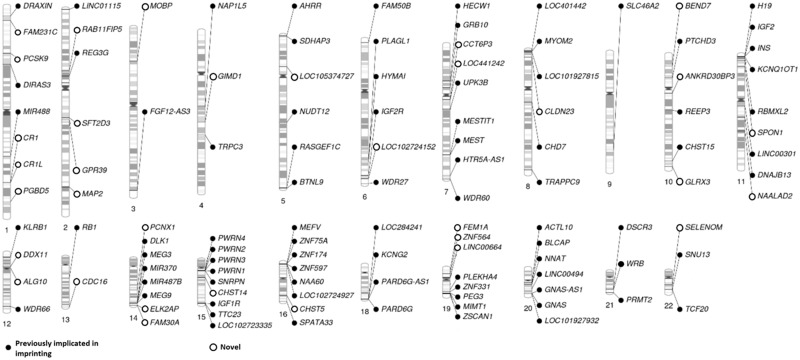
Candidate imprinted loci. Genes nearest to CpGs exhibiting statistically significant POEs. Multiple dots are shown at the same locus (e.g. CR1 and CR1L) when there were multiple CpGs within the same locus displaying POEs and closest to a different gene (refer to Table 1). Genes within regions previously implicated in imprinting are shown in black while those ones at least 2Mbp away from these regions are shown in white circles.

We identified 327 CpG sites with at least 1 SNP exerting POEs with a *P*-value less than our Bonferroni significance threshold of 3.7E−10 (Supplementary Material, Table S1). These CpG sites were distributed among 82 loci, each of which was defined to be at least 2 Mb distant from one another (Fig. 1). By inspecting RefSeq (32), geneimprint (http://www.geneimprint.com/; date last accessed January 10, 2018) and Otago imprinting (22) (http://igc.otago.ac.nz; date last accessed January 10, 2018) databases and the literature (21,30,33–40), we identified 178 loci previously implicated in genomic imprinting (each defined to be at least 2 Mb in each direction from one another) (Supplementary Material, Table S2). Of the 82 loci, we identified at genome-wide significant levels, 48 mapped to these previously implicated regions (Table 1), while 34 appeared to be novel (Table 2). Distance between each identified locus and the closest known imprinted gene is included in Supplementary Material, Table S3.

**Table 1.** CpG sites displaying POEs near known imprinted loci

Locus	Nearest gene	Chr	BP	CpG	SNP	EA	NEA	R	Birth *P*	Child. *P*	Adol. *P*	BSGS *P*	POE pattern
1	*DRAXIN*	1	11783294	cg18285337	rs4845874	G	A	0.36	2.90E−20	1.26E−20	1.32E−22	3.53E−01	U
2	*DIRAS3*	1	68516472	cg16682227	rs1430754	T	C	−0.4	1.71E−14	5.68E−17	6.57E−18	5.77E−09	U
3	*MIR488*	1	177001903	cg18865685	rs16850689	T	C	−0.31	2.68E−09	3.02E−14	8.79E−15	1.41E−07	U
4	*REG3G*	2	79220881	cg14005019	rs283842	A	C	0.4	1.49E−14	7.01E−28	4.98E−17	4.02E−15	U
5	*FGF12-AS3*	3	192289245	cg17611045	rs10460805	C	T	−0.31	2.11E−12	8.71E−13	1.96E−18	9.91E−10	U
6	*NAP1L5*	4	89619051	cg19151808	rs10428273	A	G	−0.92	0.00E+00	0.00E+00	0.00E+00	7.77E−09	B
7	*TRPC3*	4	122854405	cg16501140	rs13121031	C	G	0.26	1.84E−04	1.46E−11	3.55E−04	2.15E−06	U
8	*NUDT12*	5	102898648	cg09166085	rs7730302	T	C	0.42	2.84E−20	1.12E−20	5.00E−21	2.64E−19	U
9	*RASGEF1C*	5	179588440	cg08453205	rs10078657	A	T	0.23	8.64E−09	5.79E−12	2.28E−07	2.41E−04	U
	*BTNL9*	5	180487084	cg07774765	rs10054109	T	C	−0.26	4.01E−10	4.19E−14	2.77E−07	NA	U
10	*FAM50B*	6	3849327	cg25195497	rs2239713	T	C	0.53	5.81E−36	4.55E−73	2.00E−73	8.15E−31	U
11	*PLAGL1*	6	144329672	cg21526238	rs11155342	A	G	−0.68	4.01E−33	4.84E−50	5.11E−39	9.55E−63	U
	*HYMAI*	6	144329732	cg21952820	rs6937531	T	G	−0.67	5.98E−62	1.92E−82	4.79E−69	3.08E−42	B
12	*IGF2R*	6	160427501	cg08350488	rs8191738	A	G	−0.26	7.59E−34	2.57E−29	1.99E−17	2.95E−06	B
13	*WDR27*	6	170054730	cg19089141	rs3823464	A	G	−0.51	1.59E−37	7.08E−40	3.89E−39	2.61E−24	U
14	*HECW1*	7	43151725	cg06096382	rs10226468	C	T	−0.21	5.39E−04	2.15E−10	6.70E−11	1.64E−01	U
15	*GRB10*	7	50849639	cg09150232	rs6976501	G	A	−0.32	5.56E−01	1.19E−10	4.19E−22	1.47E−10	U
16	*UPK3B*	7	76145632	cg16453056	rs10952936	T	C	−0.31	1.54E−08	2.33E−17	1.66E−15	3.59E−06	U
17	*MESTIT1*	7	130130383	cg26275543	rs17164989	T	C	−0.41	4.74E−28	5.55E−20	1.37E−21	7.13E−12	U
	*MEST*	7	130132453	cg13986840	rs2301335	G	A	0.37	4.90E−12	2.62E−24	7.38E−19	4.37E−17	U
18	*HTR5A-AS1*	7	154863381	cg09623773	rs732050	G	A	0.24	7.23E−08	8.35E−11	3.47E−09	7.84E−07	U
19	*LOC401442*	8	832260	cg03494825	rs10110537	T	C	0.44	6.48E−13	1.03E−38	8.16E−39	2.04E−13	U
	*MYOM2*	8	2075777	cg21847720	rs2280902	A	G	−0.27	5.83E−12	3.28E−10	3.01E−13	2.08E−08	U
	*LOC101927815*	8	2591411	cg08242633	rs4875852	C	T	−0.29	7.23E−21	2.19E−18	6.68E−21	3.59E−06	U
20	*CHD7*	8	61626625	cg26441877	rs10957154	A	G	−0.24	1.43E−04	4.10E−08	9.55E−11	3.33E−04	U
21	*TRAPPC9*	8	141359539	cg26135849	rs10091104	C	T	−0.35	2.48E−22	9.60E−24	5.77E−21	5.04E−14	U
22	*SLC46A2*	9	115652824	cg07758904	rs13283782	T	C	0.36	1.11E−12	1.56E−14	2.41E−17	1.80E−13	U
**23**	***PTCHD3***	**10**	27702309	cg13458005	rs2505330	T	C	−0.2	2.63E−08	2.15E−11	1.26E−14	3.13E−09	U
24	*REEP3*	10	65733388	cg19573236	rs12570824	G	A	−0.19	1.70E−06	4.65E−11	2.86E−08	3.35E−02	U
25	*CHST15*	10	125751413	cg20250269	rs4929810	A	G	0.34	9.94E−08	4.00E−13	7.96E−14	7.32E−10	U
26	***H19***	**11**	2021103	cg27372170	rs2107425	T	C	0.51	1.05E−38	8.28E−75	1.81E−54	1.98E−18	B
	*IGF2*	11	2171694	cg25742037	rs4320932	C	T	0.33	8.88E−15	1.76E−22	1.17E−16	9.52E−05	U
	*INS*	11	2182618	cg25336198	rs3741212	A	G	−0.25	5.59E−04	9.75E−11	3.30E−05	NA	U
	*KCNQ1OT1*	11	2721591	cg09518720	rs231356	T	A	−0.6	4.91E−52	2.11E−60	3.57E−70	3.96E−38	B
27	*RBMXL2*	11	7110083	cg23916104	rs7114066	G	C	0.25	8.33E−07	4.09E−12	2.96E−09	3.39E−03	U
**28**	***LINC00301***	**11**	60414689	cg17588350	rs1994457	A	G	0.2	0.000137	1.54E−10	8.09E−12	1.76E−09	U
29	*DNAJB13*	11	73676012	cg25592907	rs605442	T	C	0.38	1.12E−21	4.40E−30	5.59E−26	9.78E−06	U
30	*WDR66*	12	122356390	cg21171335	rs10840631	C	T	−0.36	6.97E−20	5.70E−23	4.66E−19	2.97E−13	U
31	*RB1*	13	48892244	cg11408952	rs9316395	A	T	0.62	2.91E−36	3.51E−73	1.59E−61	2.22E−17	U
32	*DLK1*	14	101194748	cg18279536	rs1004573	C	G	0.63	1.62E−33	3.40E−106	5.92E−78	4.05E−18	U
	*MEG3*	14	101294147	cg08698721	rs7156824	A	C	0.39	2.00E−20	6.16E−18	1.84E−28	3.57E−14	U
	*MIR370*	14	101367300	cg16126137	rs1956128	A	T	0.39	9.89E−12	1.13E−32	1.73E−23	4.59E−07	U
	*MIR487B*	14	101512612	cg19560831	rs10083406	C	A	−0.39	2.09E−09	7.36E−45	1.68E−25	9.72E−15	U
	*MEG9*	14	101696245	cg04165845	rs17587049	A	C	−0.52	5.97E−22	3.65E−33	4.33E−41	1.12E−06	U
33	***PWRN4***	**15**	24105674	cg07956282	rs1380551	G	A	−0.15	7.06E−08	3.5E−11	1.72E−08	0.000599	U
	*PWRN2*	15	24506388	cg13749113	rs12911863	C	T	−0.61	1.34E−63	7.24E−84	1.88E−74	NA	U
	*PWRN3*	15	24672032	cg26288595	rs8033671	T	C	0.49	2.34E−13	1.24E−39	8.18E−31	NA	U
	*PWRN1*	15	24803245	cg03402443	rs6576317	A	C	0.3	2.67E−14	2.30E−16	4.58E−13	NA	U
	*SNRPN*	15	25123688	cg01786704	rs12906774	C	G	−0.45	2.01E−27	1.69E−33	3.33E−33	1.447E−08**	U
34	*IGF1R*	15	99408958	cg12553689	rs11247377	G	A	−0.37	8.97E−15	3.80E−20	2.77E−22	1.92E−14	B
	*TTC23*	15	99789855	cg16052317	rs12911333	A	T	0.38	2.90E−08	8.18E−09	1.72E−11	2.34E−03	U
	***LOC102723335***	**15**	101098829	cg02597199	rs12915921	T	C	0.1	7.23E−10	1.97E−11	6.09E−08	0.000108	U
35	*MEFV*	16	3304449	cg08260052	rs224217	G	A	0.28	6.11E−05	1.66E−09	2.73E−15	1.85E−04	U
	*ZNF75A*	16	3355951	cg04234063	rs220381	G	A	0.26	4.74E−10	9.71E−12	9.07E−07	8.63E−05	U
	*ZNF174*	16	3464107	cg01330954	rs17136367	C	G	−0.23	1.11E−03	1.03E−10	5.30E−04	1.43E−03	U
	*ZNF597*	16	3481970	cg02880119	rs171634	A	G	−0.74	5.40E−100	7.00E−162	3.60E−113	2.46E−51	U
	*NAA60*	16	3507492	cg21433313	rs1690450	A	G	−0.25	6.90E−04	8.48E−12	2.25E−07	9.89E−07	U
	*LOC102724927*	16	3988869	cg05351887	rs2531995	C	T	0.24	9.65E−08	1.01E−08	6.84E−11	5.17E−07	U
36	*SPATA33*	16	89740564	cg03605463	rs2115401	T	C	−0.58	1.02E−77	6.52E−86	1.01E−77	3.77E−26	U
37	*LOC284241*	18	77376689	cg10929690	rs3786235	T	C	−0.34	1.71E−17	6.41E−22	6.01E−23	5.56E−13	U
	*KCNG2*	18	77659695	cg05491587	rs12456484	G	C	−0.35	4.63E−19	8.67E−22	1.29E−20	NA	U
	*PARD6G-AS1*	18	77905119	cg18973878	rs11659843	T	A	−0.32	7.55E−07	5.32E−21	1.95E−07	1.12E−07	U
	*PARD6G*	18	77918588	cg07500432	rs3809927	G	C	0.27	4.31E−11	1.59E−08	2.29E−14	2.60E−11	U
38	*LINC00664*	19	21666788	cg06405146	rs2562458	G	A	−0.34	1.57E−13	7.88E−18	1.12E−13	1.50E−11	U
39	***ZNF331***	**19**	54041329	cg04522821	rs16984967	C	A	−0.32	4.12E−11	5.37E−20	4.45E−18	1.71E−47	**U**
40	*PEG3*	19	57350503	cg07310951	rs2040857	C	T	−0.3	4.19E−09	3.77E−09	2.74E−18	NA	U
	*MIMT1*	19	57376177	cg06627087	rs411808	C	T	−0.32	2.99E−16	5.72E−17	2.12E−12	5.97E−05	U
	*ZSCAN1*	19	58566643	cg18075691	rs4801552	G	A	0.28	1.09E−13	1.56E−11	2.93E−12	7.45E−07	U
41	*ACTL10*	20	32256071	cg13403462	rs6088244	T	C	−0.41	1.34E−17	4.16E−38	1.70E−25	1.84E−07	U
42	*BLCAP*	20	36148954	cg14765818	rs2064638	G	A	−0.47	7.04E−30	3.65E−55	1.29E−39	1.18E−27	U
	*NNAT*	20	36149455	cg21588305	rs2064638	G	A	−0.36	3.61E−10	1.15E−22	4.13E−15	1.33E−16	U
43	*LINC00494*	20	47013841	cg25181043	rs7267199	G	T	−0.35	4.00E−21	1.21E−21	2.33E−16	3.96E−09	U
44	*GNAS-AS1*	20	57426935	cg03606258	rs11699704	C	T	−0.86	5.10E−167	2.50E−164	2.10E−190	5.93E−80	B
	*GNAS*	20	57427146	cg24617313	rs6015389	C	T	−0.88	2.30E−284	0.00E+00	0.00E+00	1.49E−75	B
	*LOC101927932*	20	57463991	cg09885502	rs2057291	A	G	0.8	6.80E−147	2.70E−203	2.80E−161	4.86E−08	B
45	*DSCR3*	21	38630234	cg11287055	rs2051399	T	C	−0.27	1.07E−15	3.42E−11	5.71E−12	3.39E−05	U
46	*WRB*	21	40757691	cg00606841	rs2244352	T	G	0.41	9.79E−08	7.12E−23	5.73E−30	6.33E−22	U
47	*PRMT2*	21	48081686	cg24877093	rs6518306	T	C	−0.35	2.96E−19	2.66E−15	1.73E−16	6.14E−07	U
48	*SNU13*	22	42078707	cg11677105	rs4822052	A	G	0.52	8.51E−37	1.77E−60	1.81E−37	2.05E−26	U
	*TCF20*	22	42548783	cg15557168	rs2143139	G	C	−0.26	2.28E−10	7.67E−14	1.90E−10	2.67E−03	U

**Figure 2.**
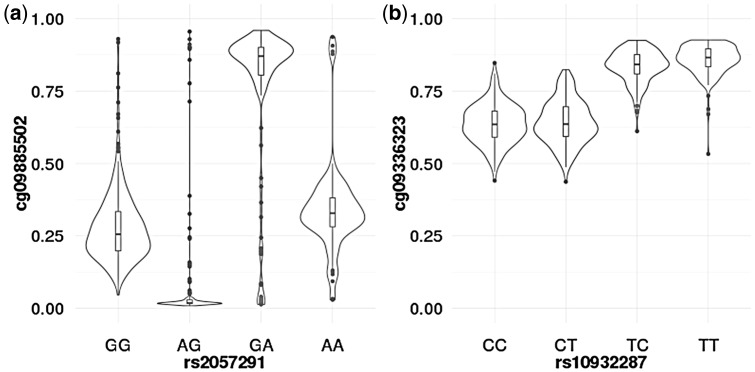
Patterns of parent-of-origin effects. Violin plots showing two patterns of CpG methylation observed in this study: (**A**) Bipolar dominance pattern observed at a CpG site in the *GNAS/GNAS-AS1* locus where one heterozygous genotype (A allele is paternally derived) has a larger mean phenotypic value than the two homozygotes and the other heterozygote (A allele is maternally derived) has a smaller mean value; (**B**) The canonical pattern of imprinting observed at a CpG site near *MAP2*, where one of the alleles leads to a larger phenotypic value than the other and one of the chromosomes is putatively silenced.

For each CpG site meeting experiment-wide significance, we show the SNP that produced the strongest P-value for the POE term. If more than one CpG site was located near the same gene, the one with the smallest *P*-value is shown. A locus is defined to be 2 Mb apart from one another. Minor alleles (MAF <50%) were used as effect alleles (EA) while the major alleles were set to non-effect alleles (NEA). Effects are summarized as partial correlations (R) between the POE coding and methylation β value at the CpG site. Parent-of-origin genotype coding was defined as −1 for heterozygotes where the minor allele was inherited from the father, 0 for homozygotes and 1 for heterozygotes where the minor allele was inherited from the mother. The gene reported is the one that is closest to the CpG site’s position. *P*-values for the POE between the CpG and the SNP are shown for each time point. In POE pattern ‘U’ refers to a uniparental effect and ‘B’ refers to a bipolar pattern. A definition of the POE patterns is illustrated in Figure 2.

** *P*-value of a proxy CpG and SNP is reported for the BSGS cohort.

CpG BP, CpG base pair position; Birth *P*, Child. *P* and Adol. *P*: *P*-value of SNP parent-of-origin effect on the CpG using DNA methylation measured at Birth, Childhood and Adolescence, respectively.

**Table 2.** CpG sites displaying POEs at least 2Mb apart from known imprinted loci

Locus	Nearest gene	Chr	BP	CpG	SNP	EA	NEA	R	Birth *P*	Child. *P*	Adol. *P*	BSGS *P*	POE pattern
1	*FAM231C*	1	17053886	cg12648811	rs1977269	A	C	0.23	7.70E−09	3.15E−10	5.82E−10	NA	U
2	*PCSK9*	1	55522104	cg13462158	rs2479418	G	A	0.42	1.03E−25	4.46E−29	1.94E−19	2.28E−06	U
**3**	***CR1***	**1**	207670014	cg00175709	rs10779362	A	T	0.19	8.71E−08	1.51E−11	0.000325	9.52E−09	U
	***CR1L***	**1**	207842833	cg03408135	rs11118410	G	A	−0.24	1.1E−09	1.67E−13	5.32E−09	4.23E−08	U
4	*PGBD5*	1	230468611	cg15363333	rs7414930	T	G	0.24	2.89E−03	3.97E−11	2.88E−07	7.04E−05	U
5	*LINC01115*	2	863946	cg01854967	rs4561699	A	G	−0.38	1.53E−13	6.72E−21	9.68E−19	2.52E−09	U
6	*RAB11FIP5*	2	73384389	cg01422370	rs6760964	G	C	−0.28	3.31E−11	3.82E−−14	3.95E−13	5.13E−04	U
7	*SFT2D3*	2	128453335	cg03738707	rs11681053	C	T	−0.23	4.58E−08	5.33E−09	2.11E−10	1.73E−03	U
8	*GPR39*	2	133402827	cg07916022	rs3738842	A	G	0.32	1.07E−11	3.15E−−19	1.31E−11	3.19E−06	U
9	*MAP2*	2	210074276	cg09336323	rs10932287	T	C	−0.73	2.00E−118	6.30E−155	2.60E−146	6.65E−25	U
10	*MOBP*	3	39543515	cg03054684	rs561543	A	G	−0.26	1.83E−06	1.58E−08	8.20E−11	1.65E−04	U
11	*GIMD1*	4	107446698	cg20025135	rs5017898	C	G	0.36	6.08E−10	6.80E−12	2.35E−09	4.47E−05	U
12	*AHRR*	5	421733	cg00976097	rs2672724	T	C	−0.25	1.77E−11	2.59E−08	3.92E−06	5.26E−02	U
	***SDHAP3***	**5**	1594676	cg21167402	rs7734561	G	A	0.26	4.66E−14	4.09E−15	6.4E−10	NA	U
13	*LOC105374727*	5	37209440	cg00331501	rs11743146	A	C	0.25	9.08E−11	7.85E−13	2.77E−07	4.31E−04	U
14	*LOC102724152*	6	164461074	cg19287610	rs7765982	T	C	−0.44	8.57E−23	1.71E−41	4.99E−38	1.92E−16	U
**15**	***CCT6P3***	**7**	64541193	cg20849893	rs10949962	G	T	0.23	4.56E−15	1.22E−18	1.21E−18	NA	U
	***LOC441242***	**7**	65235340	cg06263672	rs2418470	A	G	0.19	1.59E−12	1.58E−11	2.56E−11	8.91E−09	U
16	*WDR60*	7	158750244	cg12954512	rs6957744	A	C	−0.3	1.02E−12	2.39E−23	1.52E−13	1.05E−05	U
17	*CLDN23*	8	8559999	cg06671706	rs1060106	G	A	0.45	3.87E−28	8.10E−25	1.77E−23	1.39E−11	U
18	*BEND7*	10	13481944	cg24686497	rs11258384	G	A	0.33	6.75E−09	8.23E−10	2.19E−12	2.32E−08	U
19	*ANKRD30BP3*	10	45694889	cg26510023	rs10793594	C	A	−0.43	8.26E−28	1.98E−34	4.09E−37	1.47E−13	U
20	*GLRX3*	10	131989849	cg11372818	rs11017128	G	A	0.33	4.74E−14	7.30E−22	6.46E−17	4.48E−03	U
21	*SPON1*	11	14281011	cg02886208	rs10766125	T	C	−0.24	1.77E−09	9.30E−15	1.79E−13	3.90E−07	U
22	*NAALAD2*	11	89867911	cg14304817	rs10734123	A	G	0.32	4.39E−04	1.81E−11	4.15E−04	1.40E−01	U
23	*KLRB1*	12	9555480	cg13830619	rs10743781	T	C	0.19	6.27E−08	4.91E−11	3.77E−08	0.0019277	U
24	*DDX11*	12	31272865	cg08537890	rs11051208	G	A	0.5	8.86E−42	6.09E−43	3.08E−51	8.43E−21	U
25	*ALG10*	12	34506462	cg02590409	rs10466832	T	C	0.29	2.63E−14	2.81E−15	9.77E−11	NA	U
26	*CDC16*	13	114965839	cg12584960	rs9562157	A	G	−0.24	1.41E−10	6.50E−13	2.08E−09	4.61E−04	U
27	*PCNX1*	14	71606274	cg15816911	rs221900	T	C	−0.3	2.69E−10	4.07E−20	5.54E−12	4.48E−07	U
28	*ELK2AP*	14	106183770	cg10832239	rs4977158	G	T	0.06	3.56E−10	2.13E−11	>0.05*	NA	U
	*FAM30A*	14	106374384	cg10270204	rs17646414	T	C	−0.06	>0.05*	1.37E−10	>0.05*	NA	U
**29**	***CHST14***	**15**	40779019	cg15385345	rs11070295	T	C	−0.35	2.52E−10	0.000913	0.0000318	0.00548	U
30	*CHST5*	16	75563489	cg02390813	rs2550886	C	T	−0.24	2.49E−08	9.30E−13	6.95E−08	7.92E−08	U
31	*FEM1A*	19	4784940	cg22992730	rs3087692	A	G	−0.5	7.88E−23	6.27E−34	4.51E−38	0.0001766	U
32	*ZNF564*	19	12624832	cg01559901	rs4804712	T	G	0.28	4.75E−14	1.01E−11	1.03E−09	1.87E−07	U
33	*PLEKHA4*	19	49340593	cg26267310	rs16982311	T	C	0.37	1.63E−11	2.07E−10	2.16E−09	7.35E−05	U
34	*SELENOM*	22	31500896	cg21361322	rs11705137	C	T	−0.27	2.02E−07	3.26E−16	4.18E−10	1.01E−09	U

For each CpG site meeting experiment-wide significance, we show the SNP that produced the strongest P-value for the POE term. If more than one CpG site was located near the same gene, the one with the smallest *P*-value is shown. A locus is defined to be 2 Mb apart from one another. Minor alleles (MAF <50%) were used as effect alleles (EA) while the major alleles were set to non-effect alleles (NEA). Effects are summarized as partial correlations (R) between the POE coding and methylation β value at the CpG site. Parent-of-origin genotype coding was defined as −1 for heterozygotes where the minor allele was inherited from the father, 0 for homozygotes and 1 for heterozygotes where the minor allele was inherited from the mother. The gene reported is the one that is closest to the CpG site’s position. *P*-values for the POE between the CpG and the SNP are shown for each time point. In POE pattern ‘U’ refers to a uniparental effect and ‘B’ refers to a bipolar pattern. A definition of the POE patterns is illustrated in Figure 2.

* Results where the test of association did not reach nominal significance (P-value >0.05) were not stored.

CpG BP, CpG base pair position; Birth *P*, Child. *P* and Adol. *P*: *P*-value of SNP parent-of-origin effect on the CpG using DNA methylation measured at Birth, Childhood and Adolescence, respectively.

The POEs identified were remarkably consistent across the different time points (i.e. birth, childhood and adolescence), with 63 loci identified as statistically significant at at least two time points (i.e. *P* < 3.7E−10). All the remaining loci with the exception of the *FAM30A* locus showed at least a nominally significant parent of origin *P*-value (<0.05) between the SNP and methylation at the relevant CpG site at all three time points (Tables 1 and 2).

The strongest POEs were observed within loci previously implicated in imprinting. For instance, we observed partial correlations (*R*) as high as 0.90 between parent-of-origin coded SNPs and CpG sites near the *NAP1L5* and *GNAS* genes. For the novel candidate imprinted loci we observed partial correlations as high as *R* = 0.73 for a CpG near *MAP2*. In Supplementary Material, Tables S4–6, we have included the summary statistics of each CpG site with at least one significant SNP at each of the different time points along with additive and dominance effect statistics.

Using data from the Brisbane Systems Genetics Study (BSGS) (41,42) we tested whether each of the CpG–SNP pairs displayed in Table 1 also exhibited POEs in that cohort. We observed that 76 out of the 82 loci presented nominally significant POEs (*P*-value <0.05) in this dataset. Amongst these, 30 out of the 34 novel loci replicated in this independent cohort. The Pearson correlation between effect sizes of POEs of these 82 loci in BSGS and effect sizes from adolescents in ALSPAC was *R* = 0.8 (*P*-value =2.05E−42) (Supplementary Material, Fig. S1).

For some of the CpG sites, we observed patterns of methylation where the effect depended on the combination of the alleles (Fig. 2). For example, the distribution of DNA methylation at the CpG probe cg24617313 near the known imprinted genes *GNAS* and *GNAS-AS1* resembled a bipolar dominance pattern (6) where the phenotypic value of the two homozygotes did not differ, and one of the heterozygotes had a larger phenotypic value than the two homozygotes and the other heterozygote had a smaller value (Fig. 2A). This type of pattern was also observed for some of the CpG sites near the *NAP1L5*, *HYMAI*, *IGF2R*, *H19*, *IGF2*, *KCNQ1OT1* and *IGF1R* genes (Supplementary Material, Fig. S2). It is important to note, however, that these loci not only contained CpG sites showing bipolar dominance patterns, but also contained other CpGs exhibiting the canonical pattern (i.e. uniparental effects) of imprinting (Table 3; Supplementary Material, Figs S3–S8). For instance, at the locus containing *NAP1L5*, 7 CpG sites displayed statistically significant POEs, but only three of them resembled a bipolar dominance pattern. Most of the loci identified displayed a DNA methylation distribution consistent with uniparental effects, where one of the alleles led to a larger average phenotypic value than the other and one of the chromosomes was putatively silenced. Figure 2B shows an example of this methylation pattern, where the mean DNA methylation of the CpG probe cg09336323 near *MAP2* increases only if the minor allele ‘T’ is inherited from the father.

**Table 3.** Loci containing CpG sites displaying unusual parent-of-origin effect patterns

Locus	Bipolar dominance	Canonical	Chromosome	Range of CpG sites positions
***NAP1L5***	cg19151808	cg18607468, cg06617468, cg23954636, cg11300971, cg01174175, cg01026744	4	89, 618, 982 - 89, 619, 053
***HYMAI/PLAGL1***	cg21952820	cg08263357, cg23460430, cg11532302, cg21526238	6	144, 329, 672 - 144, 329, 789
***IGF2R***	cg08350488		6	160, 427, 501
***H19/IGF2/INS/ KCNQ1OT1***	**cg27372170**, **cg09518720**	**cg00237904**, **cg25281616**, **cg25574978**, **cg18454954**, **cg02657360**, **cg02886509**, **cg01585333**, **cg02425416**, **cg25742037**, **cg11297256**, **cg03996735**, **cg04975775**, **cg15886040**, **cg16675558**, **cg18104242**, **cg18362496**, **cg24605090**, **cg27300742, cg23476401, cg25336198**	11	2, 019, 587 - 2, 721, 591
***IGF1R/TTC23/ LOC102723335***	cg12553689	cg26163234, cg16052317, cg02597199	15	99, 408, 958 - 101098829
***GNAS***	**cg08091561**, **cg07947033**, **cg06200857**, **cg03606258**, **cg24617313**, **cg09885502**	cg04132853, cg25090051, cg00732970, cg17696847, cg23732978, cg20019489, cg02274728, cg26102503, cg06693667, cg04677683, cg15160445, cg25326570, cg23249369, cg13728472, cg20213508, cg11480267, cg03837903	20	57, 414, 039 - 57, 464, 000

Most of the loci containing a CpG site with a bipolar dominance pattern also contained CpG sites displaying a canonical pattern (i.e. uniparental effect).

### Overlap with known imprinted loci for complex traits and diseases

Previous GWAS of complex traits and diseases have reported SNPs that show parent of origin specific associations. Kong *et al*. (16) found that rs231362 showed a parent of origin specific association with T2D. In our study, this SNP displayed a similar POE (*P*-value =3.09E−12) on the CpG probe cg09518720 close to *KCNQ1OT1*. Kong *et al*. also found that the SNP rs2334499 showed a parent of origin-specific association with T2D and that the association exhibited a bipolar dominance pattern. This SNP lies 300 kb away from the *H19* locus where we also observed SNPs that show parent of origin specific associations and bipolar dominance patterns. A recent large-scale GWAS of age at menarche found that the SNP rs7141210 in the *DLK1* gene exhibited POEs. This SNP shows similar patterns in our data at the CpG site cg18279536 close to the *DLK1* gene (*P*-value =5.01E−35) (18). A recent genetic study of height found that SNPs within the IGF2-*H19* and *DLK1-MEG3* regions displayed POEs (43). However, most of the SNPs reported in that study were rare, and were thus not analysed in our study with the exception of rs7482510 where we observed a POE (*P*-value =2.81E−11) on the CpG site cg25742037 near the gene *IGF2*.

### Using methylation to determine allelic transmissions

Given that many of the loci showing parent of origin effects were associated very strongly with patterns of methylation, we were interested in the extent to which patterns of methylation might be used to determine parental transmissions in heterozygous individuals. We examined the performance of a simple statistical approach to determining transmissions at loci showing evidence of imprinting through first modelling the methylation levels of homozygous individuals, and then using this information to estimate the transmission status of each heterozygous individual (see ‘Materials and Methods’). Supplementary Material, Table S7 displays the accuracy by which the heterozygous genotypes groups could be inferred using methylation levels at the single most strongly associated CpG site at each locus. The median accuracy for discriminating between heterozygote groups for the 85 loci identified in this study was area under the receiver operator characteristic curve (AUC) =0.73 (interquartile range: 0.68–0.79) (Supplementary Material, Table S7).

Although for the majority of loci, the parental origin of alleles is difficult to determine with appreciable accuracy using DNA methylation alone, it may be the case that given very large numbers of individuals, it may still be possible to detect POEs in a large GWAS study of unrelated individuals when epigenome-wide association studies (EWAS) data are also present. In Supplementary Material, Table S7, we show the sample size that would be required to achieve 80% power to detect POEs at candidate loci (*α* = 0.0005). The sample size required increased with lower AUC and lower MAF. For example, on average, an SNP inferred with an AUC ∼0.75 and an MAF ∼0.25 required a sample size 12× larger than if the SNP was inferred with perfect discrimination (AUC =1). For more common SNPs (MAF >0.4) and AUC ∼0.75 the required sample would be 5× larger.

## Discussion

### Summary of candidate imprinted loci

In this work, we presented a genome-wide scan of SNPs’ POEs on DNA methylation from peripheral blood at multiple time points. We found that most of the POEs of SNPs on DNA methylation are constant throughout birth, childhood and adolescence. This observation is consistent with previous studies, which showed that although patterns of DNA methylation at many CpG sites in peripheral blood cells are not stable over time, the additive genetic effects of SNPs on methylation appear to be remarkably consistent longitudinally (44). We also showed that investigating POEs on DNA methylation is a powerful method of identifying candidate regions of the genome that may be affected by genomic imprinting. This assertion is supported by the fact that most statistically significant associations in our study corresponded to known imprinted loci and that the associations were with genetic variants in *cis*—i.e. it is unlikely that *cis* effects at genes are a product of maternal or paternal effects on children’s DNA methylation, as we would expect that maternal/paternal effects were distributed evenly over the genome and hence much more likely to be *trans* effects rather than *cis* effects. Interestingly we note that SNPs at the *AHRR* locus showed evidence for POEs, and these effects were strongest in cord blood (then at Age 7 years, then at Age 15 years). Methylation of CpG sites at this locus is known to be affected by smoking (45), and maternal smoking can induce changes in methylation at the same locus in offspring cord blood (46). However, it is unclear how maternal smoking could correlate with transmission of SNPs at the AHRR locus and thus produce evidence for parent of origin effects on methylation at this same locus. We also note that other mechanisms that could lead to the appearance of POEs in the absence of imprinting, and that we are unable to verify are trinucleotide expansions that are sensitive to the sex of the parent that transmits them (47,48).

Most of the loci identified in the ALSPAC dataset replicated in the BSGS. Specifically, 30 out of the 34 novel loci and 76 out of the 82 loci identified overall replicated with a *P*-value <0.05. For the nine loci where we did not observe POEs in this dataset, in the case of five, either the CpG or the SNP was missing and we did not have a proxy SNP (*R*^2^ > 0.8) to assess POEs. For the remaining four loci (CpG’s near *NAALAD2*, *AHRR*, *DRAXIN*, *HECW1*) that did not replicate, the smaller sample size of the BSGS may have impacted the results.

**Figure 3.**
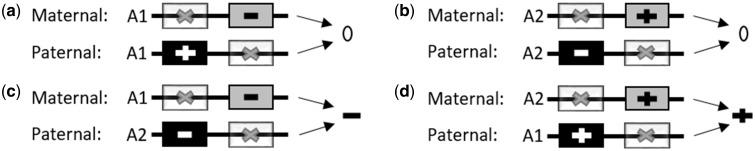
A Mechanism that generates a bipolar dominance pattern. Each of the panels in the figure displays the same two SNPs (in grey and in black) which are in high LD with each other on two different haplotypes (A1 and A2). In the case of the A1 haplotype, the allele encoded by the black SNP has a positive effect on the phenotype while the allele encoded by the grey SNP has a negative effect. In the case of the A2 haplotype, the allele encoded by the black SNP has a negative effect on the phenotype while the allele encoded by the grey SNP has a positive effect. In this example, genomic imprinting results in the black SNP being inactive in the chromosome inherited by the mother and the grey SNP being inactive in the chromosome inherited by the father. In panels (**A**) and (**B**), individuals who receive two copies of either haplotype A1 or haplotype A2 have a mean phenotype of 0. In panel (**C**) the effect on phenotype is negative as haplotype A1 is inherited from the mother and haplotype A2 from the father. In panel (**D**) the overall effect is positive as haplotype A2 is inherited from the mother and the haplotype A1 from the father.

In addition to suggesting the existence of multiple imprinted loci that have yet to be characterized, we also found multiple examples of POEs on methylation that resemble unusual imprinting patterns (Table 3). In particular, we observed bipolar dominance patterns among some CpG sites near the insulin-like growth factors and receptors *IGF1R*, *IGF2R* and *IGF2*, all of which are known imprinted loci that are located on different chromosomes. Bipolar dominance patterns have been observed previously (6,15) and are hypothesized to occur when differentially imprinted genes are in tight linkage disequilibrium (LD) but exert opposing effects on the phenotype (Fig. 3). There were also other genes nearby CpG sites that resembled bipolar dominance POEs patterns including *GNAS*, which has been previously described to encode maternal, paternal and biallelic derived proteins (49).

In our analyses, we identified 48 loci within the 178 loci previously implicated in imprinting (summarized in Supplementary Material, Table S2) and 34 outside these regions, deemed novel. The fact that we did not detect all known imprinted loci could be for various reasons, including lack of statistical power, poor coverage of CpG sites in the HM450 array, or the fact that imprinted expression is not maintained in all cell types (30), and therefore we could not detect it in peripheral blood.

The strongest POE that we identified outside known imprinted regions was on a CpG site close to the *Microtubule-Associated Protein 2* (*MAP2*) gene which plays an essential role in neurogenesis (32). Genes located near CpGs where we also detected strong POEs included *DEAD/H-Box Helicase 11* (*DDX11*) that is involved in rRNA transcription and plays a role in embryonic development (32,50). Other interesting genes close to CpGs exhibiting POEs included *MOBP*, also involved in myelination, *CR1* which mediates cellular binding to particles and immune complexes that have activated complement, and *PCSK9*, an important gene in the metabolism of plasma cholesterol (51).

### Inferring allelic transmissions in unrelated individuals

We were able to infer allelic transmissions at heterozygous individuals with moderate confidence (AUC ≥0.8) at 31 loci. For the remaining loci, however, our predictive ability appeared to be very limited. Because of the presence of winner’s curse, these figures are likely to represent an upper limit to the predictive ability of simple approaches to resolve allelic transmission. Nevertheless, our simulations indicate that in principle POEs could be detected with this information even if allelic transmission cannot be determined with certainty given very large numbers of individuals with both EWAS and GWAS. Whilst there are no cohorts of this size that have this kind of information currently, it is possible that in the future, as the cost of microarrays decrease, these sorts of studies might be feasible, particularly in large-scale population-based cohorts like the UK Biobank where GWAS is already available (52). Alternatively, it may be possible to achieve enough power by combining cohorts with both GWAS and EWAS in a meta-analysis, as is currently being done as part of the Genetics of DNA Methylation Consortium (GoDMC). We note also that whilst we have performed power calculations using information of a single CpG per SNP, it is likely that power to detect POEs could be increased further by incorporating information from adjacent correlated CpG sites and SNPs in imperfect LD.

### Strengths and limitations

To our knowledge, this is the first study to examine the evidence for POEs on human whole-genome DNA methylation. With recent technological advances and decreasing sequencing costs, the current gold standard approach to identify imprinted genes is through RNA-seq—where it is possible to quantify the expression of heterozygote alleles (28,31,53). However, this approach is still not cost-effective as it is gene expression- and SNP-dependent; thus, imprinted genes with tissue-specific expression or lacking a heterozygous exonic SNP would be missed in the very small sample sizes that are common in such studies. In addition, such studies usually require the genotyping or sequencing of parent–child trios in order to map the transmission of the alleles. In contrast, our approach uses large-scale array data on SNPs and methylation to infer the transmission of the alleles even in absence of one parental genome. This in turn allowed us to use a large sample size that provided us with greater statistical power to detect known and candidate imprinted regions, most of which were successfully replicated in an independent dataset.

Our finding of significant POEs is less likely to be explained by experimental artefacts. In contrast to traditional GWAS that test SNPs’ additive effects in, e.g. a complex disease, where batch effects during genotyping may correlate with disease status, these should not correlate with (i) parental origin of the alleles and (ii) a quantitative trait such as DNA methylation. Similarly, batch effects during DNA methylation measurement and SNPs in the probe sequences that affect hybridization to the methylation array are not expected to correlate with parental transmission of genotypes. For example, in EWAS caution is recommended for cross-reactive probes (54) as these may lead to confounded findings (e.g. the association between methylation at a CpG site and a trait is the result of an association with another CpG site with a similar probe sequence). In the case of our study, measurement errors arising from these probes would distribute evenly between the heterozygote groups, as the microarray platform cannot distinguish between maternal and paternal transmissions. In addition, we are testing parent-of-origin effects of SNPs in *cis* to the probe and so we believe it is unlikely that the effect we see may be between the SNP and a faraway probe with a similar sequence. Nevertheless, we have removed probes that may map to other positions in the genome (54) and caution that our results, especially those at novel loci, require replication using another technology such as pyrosequencing before artefacts of the technology can be ruled out as an explanation for significant POEs.

Our approach, however, does have its weaknesses. First, we were unable to assess directly whether the identified POEs affect the expression of the genes mentioned in this study. This is particularly problematic for the novel candidate imprinted loci where there is no prior functional work to back up our assertion. The 33 novel loci found in our study were not identified in a previous large systematic analysis of imprinting across cell lines using RNA-seq (30). Nevertheless, in the latter study only 42 out of over 100 known imprinted genes were identified. There are multiple reasons to explain the lack of support of these novel loci including sub-optimal coverage and lack of power in other studies as well as the possibility that although we observe parent-of-origin DNA methylation differences, these may not translate into differences in gene expression.

The other important limitation is that we were not able to distinguish whether the allele inherited from the father or the mother is active or inactive (i.e. whether the maternal or paternal gene is silenced) as the POEs are relative, and DNA methylation seldom has a baseline of zero. For instance, taking Figure 2B as an example, we cannot distinguish between whether the DNA methylation baseline is ∼0.65 and the maternally inherited minor allele increases DNA methylation while the paternally derived allele remains inactive or vice versa.

## Conclusion

In conclusion, we report 34 novel genomic loci that exhibit parent of origin effects and consequently may be imprinted. We also show that the pattern of association at these loci remains stable from birth to adolescence. Although our approach does not replace traditional methods to detect genes subjected to imprinting, it is a convenient and cost-effective way to narrow down the search space and prioritize candidates. Consistent with what it is known about the biological role of imprinting, many of the identified loci were within or nearby genes with known effects on traits related to growth, development and behaviour. Our results require replication using another technology (e.g. pyrosequencing) and further functional experiments to confirm that such effects arise through a genomic imprinting mechanism.

## Materials and Methods

### Data

#### Study sample

ALSPAC is a geographically based UK cohort that recruited pregnant women residing in Avon (South West England) with an expected date of delivery between 1 April 1991 and 31 December 1992. A total of 15 247 pregnancies were enrolled, with 14 775 children born (55,56). Of these births, 14 701 children were alive at 12 months. Ethical approval was obtained from the ALSPAC Law and Ethics committee and the local research ethics committees. Appropriate consent was obtained from the participants for genetic analysis. Please note that the study website contains details of all the data that are available through a fully searchable data dictionary (http://www.bris.ac.uk/alspac/researchers/data-access/data-dictionary/).

The data used in this study correspond to the mother–child pairs from the ALSPAC cohort who took part in the Accessible Resource for Integrative Epigenomic Studies (ARIES, http://www.ariesepigenomics.org.uk/) (44,57). We used genotypic data from 740 mother–child duos, and DNA methylation data from the 740 children. Each child had DNA methylation measured at three time points—i.e. cord blood, peripheral blood (whole blood, buffy coats, white blood cells or blood spots) during childhood (∼7 years) and during adolescence (15 and 17 years).

#### DNA methylation

Description of the DNA methylation assays can be found elsewhere (44,57). In brief, genome-wide methylation was measured using the Illumina Infinium HumanMethylation450 (HM450) arrays. These arrays were scanned using Illumina iScan, and the initial quality review was done in GenomeStudio. A wide range of batch variables were measured for each sample during the data generation, including quality control (QC) metrics from the standard control probes on the array. Samples failing QC were not included in the analysis. Data points with a low signal: noise ratio (detection *P* > 0.01) or with methylated or unmethylated read counts of zero were also excluded from analysis. Genotype probes in the HM450 array of the same individual at different time points were used to identify and remove sample mismatches. DNA methylation at each CpG probe was normalised using the Touleimat and Tost algorithm implemented in the R package watermelon (58) to reduce the non-biological differences between probes. We removed 30 970 CpG sites with probe sequences that substantially overlapped with other locations of the genome (54). Finally, *β* values (i.e. the proportion of methylation) of 437 542 CpG sites were included in the analysis.

#### Genotypes

Mother–child duos participating in ARIES were previously genotyped as part of a former ALSPAC study, the details of which can be found elsewhere (55,56,59). Briefly, children were genotyped on Illumina HumanHap550 quad-chip platforms by the Wellcome Trust Sanger Institute (Cambridge, UK) and by the Laboratory Corporation of America (Burlington, USA) using support from 23andMe. Mothers were genotyped on Illumina HumanHap660W quad-chip platform by Centre National de Génotypage (Évry, FR). Standard QC was applied to SNPs and individuals. Individuals were excluded based on genotype rate (<5%), sex mismatch, high heterozygosity and cryptic relatedness [defined as identity-by-descent (IBD) >0.125]. In order to remove individuals of non-European descent, principal components (PCs) were derived from LD-pruned SNPs with MAF >0.01 using plink (60). Individuals laying 5 SD beyond the 1000 Genomes European population PCs 1 and 2 centroid were excluded. SNPs with a minor allele frequency (MAF) <1%, genotyping rate <5% or with a deviation from Hardy–Weinberg disequilibrium ($pP\;<<\;1\times 10^{-6}$) were removed from the analysis.

Genotype Imputation was performed by first phasing the genotypes using SHAPEIT V2 (61), and then imputing to the HapMap CEU reference panel using Impute (v2.2.2) (62). Genotypes were removed if they deviated from Hardy–Weinberg equilibrium *P* < 5 × 10^−6^, MAF <5% (the high threshold was to minimize the possibility of low frequency variants producing chance parent of origin effects through statistical fluctuation) or imputation info score <0.8. Best guess genotypes were used for subsequent analyses. The final imputed dataset used for the analyses presented here contained 2 158 724 SNPs.

### Statistical analysis

#### Identifying transmission of the alleles

The crucial first step in identifying POEs is assigning alleles to their parental origin. In order to achieve this, we applied the duoHMM algorithm implemented in the software SHAPEIT V2 (63) to the most likely imputed genotypes from the ALSPAC mothers and children. This algorithm leverages LD and IBD sharing in order to phase genotypes and resolve the parental origin of alleles at each SNP. Using a custom written *Perl* script, the phased genotypes were formatted in a way such that heterozygotes where the minor allele was inherited from the mother were coded as 1, homozygotes were coded as 0 and heterozygotes where the minor allele was transmitted by the father were coded as −1. In order to confirm the accuracy of our approach to resolve the transmission of the alleles, we compared the haplotypes of the mothers and children. We observed that for each of the children, the alleles of the haplotype inferred to be the one inherited from the mother, matched to those from the mother 99.9% of the time. We attribute the 0.1% of mismatches to genotyping or imputation errors in mothers or children. This calculation assumes that phasing is 100% accurate whereas in reality there will be some errors in the haplotyping process. We note that the accuracy of phasing is extremely high when trio data is available (i.e. >99.8%; 64) and high when using thousands of unrelated individuals with dense genotyping (>98%; 65). We expect that the accuracy of phasing using mother-offspring duos is intermediate between the two and thus enabling highly accurate determination of parent of origin information. It is also important to realize that any errors in phasing will decrease power to detect POEs, but would not lead to increased Type 1 error rates. 

### Regression model

In order to identify SNPs in the genome displaying POEs on DNA methylation from the three time points (birth, childhood and adolescence), we employed a regression model (6,66) to estimate: the additive effect *β*_A_, defined as the equal contribution of each minor allele to the phenotype; (ii) the dominance effect $\beta_{D}$ that measures the deviation of the heterozygote from the mean phenotypic value of the two homozygotes and the parent-of-origin effect $\beta_{p}$, which is the mean difference between heterozygotes (i.e. the heterozygote where allele ‘A’ is paternally transmitted, and the heterozygote where allele ‘A’ is maternally transmitted). In matrix annotation, with intercept term $\beta_{0}$, the mean phenotypic value for each possible genotype can be modelled as:

AAAaaAaa=10001111111-11200β0βAβDβP

With the genotypes (AA, Aa, aA, aa) ordered (e.g. paternal first then maternal). This coding of genotypes enables testing for effects that are strictly owing to parent-of-origin effects, as under Hardy–Weinberg equilibrium the parent-of-origin vectors are orthogonal to the additive and dominant effects.

Given that DNA methylation is affected by sex and age, these factors were incorporated into the model as covariates, along with the first three ancestry informative PCs derived from genome-wide SNP genotypes in order to control for the population stratification, as well as the first 10 PCs derived from the control matrix of the Illumina HumanMethylation450 assays to control for batch effects. The following model:

$$CpG=\beta_{0}+\beta_{A}A+\;\beta_{D}D+\;\beta_{P}P+\sum_{i=1}^{\#cov}\beta_{i}{cov}_{i}+\epsilon$$

was fitted to the 468 512 DNA methylation CpG probes against SNPs within 500 kb from the CpG probe (i.e. SNPs in *cis*). SNPs beyond 500 kb from the CpG site were not assessed as it would have increased the multiple testing burden by three orders of magnitude and the number of individuals in this study may not yield enough power to detect reliable associations of SNPs in *trans* (44). In this model, CpG is the column vector of DNA methylation values of a CpG probe; $\beta_{0}$ is the intercept; $\beta_{A}$ the regression coefficient of the SNP additive effect; *A* is the vector of genotypes in additive coding; $\beta_{D}$ the regression coefficient of the SNP dominance effect; D is the vector of genotypes in dominance coding; $\beta_{P}$is the regression coefficient of the SNP parent-of-origin effect; ***P*** is the vector of genotypes in parent-of-origin coding; $\beta_{i}$ the regression coefficient of the covariates; and cov_*i*_ are the covariates specified above.

Given that DNA methylation values suffer from heteroscedasticity, White–Huber standard errors (67) were computed to estimate the significance of the POE term $\beta_{P}$ using the *sandwich* package in R. Partial correlations displayed in Table 1 correspond to the Pearson correlation between residuals of the CpG’s DNA methylation after adjusting by the covariates described above and the parent-of-origin coded SNP.

### Significance threshold

In total, ∼400 M statistical tests were performed. Given that neighbouring SNPs usually display a high degree of correlation between each other owing to LD, the number of independent tests was empirically estimated using a matrix spectral decomposition algorithm of the correlation matrix (68,69). We applied this algorithm in 100 randomly selected autosomal genomic regions of 1 Mb each and observed that the number of independent SNPs was 0.33 times (95% CI 0.28, 0.38) the number of SNPs tested. Hence the effective number of tests was ∼132 M and the Bonferroni significance threshold was set at *P*-value <3.7E−10. We note, however, that this threshold may still be conservative as the correlation between CpG probes has not been taken into account.

### Replication

We used data from the Brisbane Systems Genetic Study (BSGS) (41,42) as replication sample. We employed a subset of 462 individuals from 176 families with genotypic and DNA methylation data where we were able to infer the parental transmission of the alleles. Detailed information about the BSGS can be found elsewhere (41,42). In brief, the participants were genotyped using the Illumina 610-Quad Beadchip and imputed against 1000 Genomes European ancestry population. Whole blood DNA methylation levels were measured with the Illumina HumanMethylation450 array and normalized as describe in McRae *et al.* (41).

Parental transmission of the alleles was inferred using the duoHMM algorithm implemented in SHAPEIT v2. A linear mixed model was fitted between each CpG–SNP pair exhibiting a statistically significant POE in the ALSPAC data shown in Table 1. For CpG–SNP pairs where the SNP was not available, a proxy SNP (*R*^2^ > 0.8) or a nearby CpG was used instead. An additive genetic relationship matrix derived from common SNPs (MAF >0.05) was employed as random effect in the linear mixed model to control for the relatedness of the individuals. Sex, age, top five PCs derived from the DNA methylation data and top two PCs derived from the genotype data were used as fixed effects.

The mean age of the BSGS cohort was 13.8 (SD* *=2.06) and thus we compared effect sizes from POEs with the ones estimated using DNA methylation data of adolescents from ALSPAC using a Pearson correlation (Supplementary Material, Fig. S1).

### Predicting parental transmission in heterozygote individuals using methylation status

During this project, we observed that DNA methylation at some CpG sites could potentially be used to infer the parental transmission in heterozygote individuals of samples without parental genotypes. Under a uniparental expression pattern of imprinting, one of the parental alleles remains inactive leading to the phenotypic mean of one of the heterozygote groups (e.g. minor allele inherited by the mother) being equal to the mean of the minor allele homozygote, while the phenotypic mean of the other heterozygote group (e.g. minor allele inherited by the father) is equal to the mean of the major allele homozygote. With this premise, we fitted a logistic model to the homozygous individuals for each of the statistically significant SNPs found in this study:

$$logit\left(H\right)=\beta_{0}+\;\beta CpG+\;\varepsilon$$

where ***H*** is a vector with labels 0 for minor allele homozygotes and 1 for major allele homozygotes and ***CpG*** is the DNA methylation at the relevant CpG site.

We then used this fitted logistic model to predict the pattern of allelic transmission for each heterozygote individual at the putatively imprinted SNPs. Note that this approach can also predict the allelic transmission at other patterns of imprinting (e.g. bipolar or polar dominance) as it splits heterozygote individuals into those that are above the phenotypic mean of the (e.g. minor allele) homozygous individuals and those that are below the phenotypic mean of the (e.g. major allele) homozygous individuals. To measure how well this method performed, we computed the Area Under the receiver operating Characteristic curve (AUC) for each SNP.

We estimated the sample size that would be required to achieve 80% statistical power to detect POEs using this approach to infer parental transmission compared with having actual parental genotypes and being able to identify each heterozygote group correctly (as was the case in this study). We simulated 500 runs for each SNP where POEs explained: 0.5, 1, 2, 4 and 9% of the variance (*R*^2^) using known parent-of-origin coded genotypes (i.e. 0 for homozygotes, and −1 or 1 for each of the heterozygote groups, AUC = 1). We then estimated how the variance explained degraded when using the inferred genotypes coded as 0 for homozygotes and as an expected dosage for heterozygotes: *P *− (1* *−*P*), where *P* is the probability of being in heterozygous group 1 and 1* *−*P* the probability of being in heterozygous group 2. For example, when we simulated a POE using the known parent-of-origin coded genotypes (i.e. AUC = 1) that explained *R*^2^ = 1%, the variance explained would drop to *R*^2^ = 0.09% when using the inferred (AUC = 0.75) parent-of-origin coded genotypes (as expected, *R*^2^ would normally degrade relative to AUC and MAF). We then used the function pwr.r.test from the ‘pwr’ package in R that implements a Z’ transformation of the correlation (70) to derive the sample size required to achieve 80% power with *α* = 0.0005.

## Supplementary Material

Supplementary Material is available at *HMG* online.

## Supplementary Material

Supplementary Data
